# Ultrasound-responsive microbubbles in refractory infection niches: multi-front targeting of microenvironmental tolerance and local delivery of toxicity-limited antibiotics

**DOI:** 10.3389/fcimb.2026.1756357

**Published:** 2026-02-11

**Authors:** Maxwel Adriano Abegg

**Affiliations:** Graduate Program in Sciences, Technology and Health (PPGCTS), Federal University of Amazonas (UFAM), Itacoatiara, Brazil

**Keywords:** antimicrobial resistance, biofilm-associated tolerance, local antibiotic delivery, microenvironment reconditioning, refractory infection niches, salvage therapy, toxicity-limited antibiotics, ultrasound-responsive microbubbles

## Introduction

Bacterial antimicrobial resistance (AMR) contributed to nearly five million deaths in 2019, with a substantial fraction directly attributable to resistant infections (Antimicrobial Resistance Collaborators, 2022). Mortality remains high in refractory cases involving carbapenem-resistant Enterobacterales and other high-risk Gram-negative pathogens despite optimized combinations (Sheu et al., 2019; Prayag et al., 2023), while the antibiotic pipeline remains fragile and multidrug-, extensively drug-, and pan-drug-resistant organisms proliferate (Makabenta et al., 2021).

Many chronic and device-related infections are now framed as microenvironmental diseases. Biofilm-associated tolerance, steep pH and oxygen gradients, and spatially structured immune responses jointly drive treatment failure, allowing bacteria to tolerate concentrations far above planktonic MICs—particularly in acidic, hypoxic niches enriched in persisters (Stewart, 2015; Kolpen et al., 2016; Behbahani et al., 2022; Bjarnsholt et al., 2022). High cell density can further promote density-dependent persistence, reducing quinolone efficacy even when MICs appear favorable (Gutierrez et al., 2017).

Nanomaterial-based therapeutics and functional dressings increasingly incorporate microenvironment-aware design. However, these can lose performance under pathophysiological conditions, requiring deliberate modulation of local pH, oxygenation, or redox state to restore activity (Hu et al., 2020; He et al., 2023; Qiao et al., 2024; Pranantyo et al., 2024). In this Opinion article, I explore how ultrasound-responsive microbubbles (MBs) could reshape this landscape. MBs have evolved from purely diagnostic contrast agents into theranostic carriers coupling real-time imaging with locally triggered release (Ferrara et al., 2007; Frinking et al., 2020; Navarro-Becerra and Borden, 2023). By analogy to regional chemotherapy, refractory infection niches could potentially be attacked on several fronts by co-localizing microenvironment modifiers, adjuvants, immune modulators, and antibiotics in synergistic regimens. MBs are well suited to probe such synergy because they can be visualized, concentrated, and triggered to co-localize effects in defined anatomic regions. A central aim here is to examine the engineering, dosing, and safety constraints required to turn this theranostic promise into a credible strategy for refractory infectious diseases.

## What ultrasound-responsive microbubbles already offer in infectious models

Clinically approved MBs such as SonoVue and Definity are gas-filled, lipid- or protein-shelled spheres (1–10 μm) that resonate under diagnostic ultrasound for vascular imaging (Ferrara et al., 2007; Frinking et al., 2020). Acoustic pressures can induce stable cavitation and microstreaming or, at higher mechanical indices, inertial cavitation with bubble collapse and strong shear forces, with shell mechanics playing a major role in acoustic behavior (Borden et al., 2006; Roovers et al., 2019).

In oncology, ultrasound-targeted microbubble destruction (UTMD) modulates the microenvironment, enhances vascular permeability, and improves immunotherapy delivery (Liu et al., 2023; Wu et al., 2023; Zheng et al., 2023). Similar principles appear in infection models as “sonobactericide”—ultrasound-based strategies combining MBs or phase-change agents with antibiotics to disrupt biofilms and potentiate killing (Lattwein et al., 2020; Durham et al., 2021; Choi et al., 2025; Liu et al., 2024). Across planktonic, biofilm, and intracellular models, ultrasound with MBs increases antibiotic efficacy via matrix weakening, enhanced penetration, and transient permeability changes (Zhu et al., 2014; He et al., 2011; Hu et al., 2018; Horsley et al., 2019; Plazonic et al., 2022; Xiu et al., 2023; Zhao et al., 2023; Chen et al., 2025). Recent work clarified the physical determinants of biofilm dispersal: at 1.1 MHz and 2500 kPa peak negative pressure, lipid-shelled microbubbles achieved near-complete dispersal (94 ± 2%) of Staphylococcus aureus biofilms, with high-speed imaging revealing that translational motion of larger microbubbles (>10 μm) across the biofilm surface—rather than inertial cavitation of smaller bubbles—was the dominant mechanism (Batchelor et al., 2025). Beyond enhanced penetration, ultrasound with microbubbles potentiates vancomycin activity against Staphylococcus epidermidis biofilms through mechanical and biochemical mechanisms, including modulation of biofilm-associated gene expression—downregulating *icaA* (matrix synthesis) while upregulating quorum-sensing regulators *agrB* and *RNAIII* that promote dispersal (Dong et al., 2017). Recent reviews highlight both promise and safety concerns, noting that most data remain preclinical with variable effect sizes (Liu et al., 2024).

Collectively, these data support a cautious hypothesis: MB-enabled cavitation can serve as a site-confirmed, pulse-like delivery modality synchronizing mechanical action and chemical exposure at refractory foci, but only within dose, anatomic, acoustic, and safety constraints.

## Positioning MBs among emerging antibacterial strategies

Because MB-based platforms can appear “all-in-one,” situating them alongside other emerging antibacterial strategies is useful. For planktonic or disseminated infections, personalized bacteriophage therapy shows encouraging outcomes as an antibiotic adjunct (Pirnay et al., 2024). For biofilm disease, advanced nanocarriers combine bactericidal and host-modulating effects, including fusidic acid–encapsulated polymeric nanoparticles (Chang et al., 2024) and antibiofilm hydrogels that disrupt EPS and restore antibiotic sensitivity (Pranantyo et al., 2024; Qiao et al., 2024).

Ultrasound can also potentiate antibiotics without pre-formed MBs. Low-frequency ultrasound improves antibiotic performance in biofilms through bioacoustic effects and altered diffusivity but lacks inherent “see-and-treat” capability (Nahum et al., 2024). *In vivo*, low-frequency ultrasound has been explored with anti-MRSA agents in pulmonary infection models (Yan et al., 2024). Ultrasound-triggered nanoscale systems—nanodroplets and phase-change nanoparticles—offer “on-demand” activation without micron-scale cavitation nuclei (Xin et al., 2022; Choi et al., 2025), while sonodynamic strategies further broaden ultrasound-enabled biofilm eradication without requiring MBs (Xin et al., 2024).

Against this backdrop, MBs retain three distinctive advantages: (i) real-time imaging to confirm localization and guide insonation; (ii) spatiotemporally controlled cavitation for mechanical disruption and transient barrier permeabilization; and (iii) conditional release of potent cargos only after imaging-guided accumulation. Their constraints—limited cargo capacity, rapid clearance, ultrasound accessibility, and acoustic heterogeneity—shape realistic development paths.

## Niche-directed microbubble single-cargo cocktails: a salvage-oriented proposal

**Figure 1:**
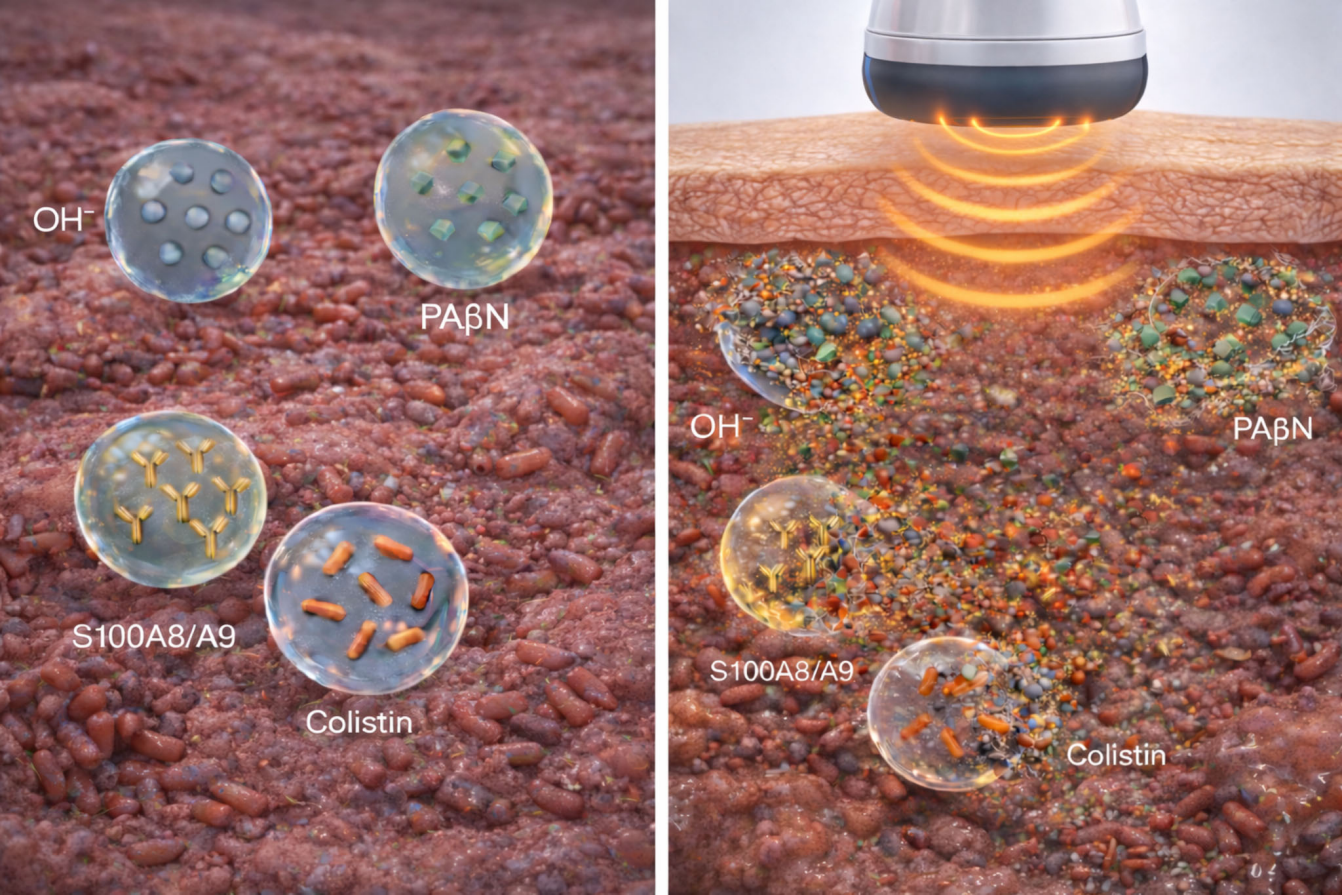
Conceptual niche-directed use of ultrasound-responsive microbubbles (MBs) to recondition refractory, infection-colonized foci and locally deliver potentiated, toxicity-limited antibiotics. Left panel: a four-population, single-cargo MB framework distributed within an infection-colonized focus, comprising microenvironment-modifying MBs releasing alkalinizing species (OH^-^), adjuvant MBs carrying an efflux-pump inhibitor (PAβN), immune-activating MBs delivering an immunomodulator (S100A8/A9), and antibiotic MBs containing a systemically constrained agent (e.g., colistin). Right panel: a focused ultrasound pulse induces cavitation and shell disruption, producing a brief, spatially confined “therapeutic pulse” that co-localizes microenvironment correction, adjuvant activity, immune engagement, and intensified local antibiotic exposure at the colonized site (including matrix-embedded or structured compartments when present). This schematic represents a conceptual upper-limit configuration; near-term translation would likely prioritize simpler combinations.

If refractory infections are treated as structured microenvironments, local therapy can be framed as coordinated attacks on multiple tolerance layers. As a boundary-mapping exercise, I propose a four-population, single-cargo MB concept where each formulation carries one functional load intended to co-localize at the infectious focus under ultrasound guidance (**Figure 1**).

First, microenvironment-modifying MBs would target local acidity or hypoxia impairing antibiotics and phagocytes (Behbahani et al., 2022; Kolpen et al., 2016; Hu et al., 2020; He et al., 2023). Second, adjuvant MBs could deliver one potentiator class (e.g., efflux inhibitors, membrane-active agents, quorum-sensing blockers, matrix dispersants) to erode biofilm tolerance and improve penetration, aligning with antibiofilm frameworks (Stewart, 2015; Bjarnsholt et al., 2022) and antibiotic-adjuvant literature (Dhanda et al., 2023; Duffey et al., 2024; Kincses et al., 2025). Third, immune-modulating MBs—the most speculative front—would require signals reducing inflammation while preserving clearance, drawing on UTMD-enabled immunomodulation workflows (Ferrara et al., 2007; Bouakaz and Escoffre, 2024; Liu et al., 2023; Wu et al., 2023) and data showing synergistic effects of immunomodulatory S100A8/A9 combined with ciprofloxacin against Pseudomonas aeruginosa biofilms in murine wound models (Laulund et al., 2019). Notably, ultrasound–microbubble treatment has demonstrated enhanced neutrophil responses against biofilms, including increased phagocytosis (~1.4-fold), oxidative burst activity, and chemotaxis (~2.5-fold), suggesting that cavitation itself may partially restore innate immune access to otherwise protected niches (Dong et al., 2017). Fourth, antibiotic MBs would deliver a focused antibacterial pulse as shell-incorporated drugs, surface-decorated molecules, or payloads in attached nanoparticles and liposomes, potentially including toxicity-limited agents once imaging confirms containment (Ferrara et al., 2007; Navarro-Becerra and Borden, 2023; Chen et al., 2019). Proof-of-concept exists with polymyxin B: chitosan-modified polymyxin B-loaded liposomes combined with ultrasound microbubbles achieved near-complete eradication of multidrug-resistant Acinetobacter baumannii biofilms at sub-MIC concentrations (2 μg/mL), representing a 16-fold reduction compared to free drug (Fu et al., 2019).

A practical question is how predominantly intravascular MBs might influence tissue-embedded infections. A meaningful fraction of tissue infections remains vascularized at their margins, where cavitation-driven microstreaming, transient endothelial disruptions, and permeability increases concentrate transport effects (Price et al., 1998; Skyba et al., 1998; Stieger et al., 2007; Lentacker et al., 2014). Even if effects are strongest near perfused boundaries, repeated image-guided insonation could generate edge-focused pulses improving penetration; conversely, effects should be weakest in necrotic or avascular cores, making lesion geometry and perfusion status central to patient selection.

In practice, this maps to salvage therapy: after image confirmation, short ultrasound bursts generate a spatially confined therapeutic pulse combining mechanical disruption and local release. An especially relevant application involves MBs ferrying agents too toxic for systemic use, releasing them only upon adequate localization—potentially rehabilitating compounds excluded due to systemic toxicity and adding functionally “new” antibiotics for extreme conditions. The logic parallels regional chemotherapy, where high local doses may be justified in patients at imminent risk of death (Sheu et al., 2019; Prayag et al., 2023).

## Technical and safety considerations

Implementing a four-component strategy introduces constraints beyond formulation stability. Moving from theory to viable salvage therapy requires confronting at least three hurdles: acoustic heterogeneity, stoichiometric limitations, and temporal mismatch between drug release, bacterial resuscitation, and host responses.

Acoustic heterogeneity and coordinated release represent the most immediate barrier. Acoustic resonance depends on bubble size and shell viscoelastic properties. Lipid-shelled, hybrid lipid–polymer, and polymer-stabilized oxygen bubbles possess distinct stiffness and damping coefficients, leading to divergent cavitation thresholds and release behaviors (Borden et al., 2006; Roovers et al., 2019; Navarro-Becerra and Borden, 2023; Chen et al., 2019). Insonation at a single frequency might trigger inertial cavitation in one group while leaving others oscillating stably. To avoid this “spectral segregation,” formulation engineers may need to reduce cocktail complexity and enforce convergent shell architectures.

Stoichiometry and the “wake-up” lag further constrain expectations. Typical contrast regimens deliver 10^8^–10^9^ MBs per mL, corresponding to only a few microliters of gas intravascularly—insufficient for bulk neutralization of acidic, buffered biofilm matrix or high agent concentrations over large tissue volumes. Microenvironment modification must be viewed as an interfacial effect, although repeated pulses might drive meaningful local adjustments (Lentacker et al., 2014; Kooiman et al., 2020). Direct *in vivo* measurement of pH or oxygenation shifts from MB cavitation in infection models remains sparse; permeability changes and enhanced tracer extravasation are more commonly reported (Price et al., 1998; Skyba et al., 1998; Stieger et al., 2007), while local pO_2_ or pH have been more extensively characterized in oxygen-releasing biomaterial studies without MBs (Hu et al., 2020; He et al., 2023)—an important knowledge gap. Moreover, bacterial resuscitation from persister states can take minutes to hours (Gutierrez et al., 2017); a transient pulse may dissipate before pathogens exit dormancy, motivating careful timing relative to peak antibiotic exposure and repeated insonation cycles.

Practical boundaries are dose- and geometry-limited. The realistic target is a boundary layer where cavitation-driven microstreaming transiently increases transport at the biofilm–liquid interface. Permeability and penetration are the most direct endpoints (Price et al., 1998; Skyba et al., 1998; Lentacker et al., 2014), and microenvironment shifts should be modest and short-lived. Cavitation effects are typically transient (minutes to hours) and spatially confined (perivascular, sub-millimeter), implying repeated insonation cycles synchronized with peak antibiotic exposure (Lentacker et al., 2014; Kooiman et al., 2020).

Ultrasound access is anatomy-dependent, favoring accessible soft tissues (Ferrara et al., 2007; Frinking et al., 2020). Although approved MBs are largely intravascular, inertial cavitation can induce transient endothelial disruptions and enhance extravasation (Price et al., 1998; Skyba et al., 1998; Stieger et al., 2007).

Immunological safety is a cross-cutting concern. Patients needing salvage therapy are often pre-septic with primed immune systems; localized release of potent agonists could precipitate tissue necrosis or systemic inflammatory escalation. Safety profiling must quantify endothelial activation, systemic spillover, and complement activation, recognizing that several shell materials can trigger complement-mediated pseudo-allergy (Ferrara et al., 2007; Frinking et al., 2020). The microbubble volume dose places a hard ceiling on total cargo; dividing limited capacity among four components is difficult to justify in early translation. Simplified single- or dual-function systems combining mechanical disruption with one chemical modality appear more technically defendable.

## From concept to plausible development path

To my best searches, to date, no infection studies explicitly combine two or more distinct single-cargo MB formulations as a coordinated multi-front regimen; most reports optimize one platform at a time (Durham et al., 2021; Plazonic et al., 2022; Li et al., 2024; Ma et al., 2025; Mu et al., 2025; Li et al., 2025). This gap reflects practical barriers: co-manufacturing, matching pharmacokinetics, aligning acoustic thresholds across heterogeneous shells, and managing safety and regulatory complexity.

In the near term, the most credible trajectory is conservative. Priority should go to reproducible single-cargo MB systems that deliver a toxicity-limited antibiotic or a single microenvironmental or adjuvant modality under image guidance, with rigorous *in vivo* testing in well-characterized models and careful measurement of both therapeutic gains and unintended vascular or immunological injury (Lattwein et al., 2020; Liu et al., 2024). Only once such platforms are technically mature would it become reasonable to test more ambitious regimens that deliver coordinated pulses from two or more single-cargo MB populations within the same infectious focus.

A more speculative, long-term path involves reducing acoustic heterogeneity through convergent shell architectures. Lipid–polymer hybrid shells with cross-linked matrices could limit phase separation, support targeted ligands, and host mechanosensitive linkers, allowing matrix-active enzymes, adjuvants, antibiotics, and immune agonists to be co-loaded into a single MB type (Borden et al., 2006; Roovers et al., 2019; Chen et al., 2019; Zheng et al., 2023; Navarro-Becerra and Borden, 2023). Systematic mapping of cavitation thresholds, release profiles, and safety in infection models would be required before clinical translation could be credibly discussed.

A plausible development path is staged: (i) establish robust single-cargo platforms in infection-relevant models; (ii) test two- and three-front combinations under shared insonation protocols with simple readouts; and only then (iii) explore higher-order combinations or convergent shell architectures. The central claim is modest but testable: in ultrasound-accessible refractory foci, MB cavitation may enable a controlled therapeutic pulse overcoming microenvironment-driven tolerance and expanding the usable space of toxicity-limited antibacterials.

## Outlook and concluding remarks

Evidence from biofilm biology, infectious disease pathology, and biomaterials science indicates that many recalcitrant infections are problems of local context—microenvironment, pathogen phenotype, and host response—rather than solely drug choice or dosing failures (Stewart, 2015; Bjarnsholt et al., 2022). Work on biofilm tolerance, microenvironment-targeted nanotechnologies, adjuvants, and sonobactericide shows that partial correction of acidity/hypoxia, efflux inhibition, biofilm disruption, and improved penetration can resensitize bacteria (Kolpen et al., 2016; Hu et al., 2020; Lattwein et al., 2020; He et al., 2023, 2011; Qiao et al., 2024; Xiu et al., 2023; Zhao et al., 2023; Liu et al., 2024). Ultrasound-responsive microbubbles occupy a distinctive position because they couple real-time imaging, physical biofilm disruption, and drug delivery (Zhu et al., 2014; Horsley et al., 2019; Xiu et al., 2023; Zhao et al., 2023).

The central argument is not that complex multi-component cocktails are clinically ready, but that articulating how far niche-directed, ultrasound-guided strategies might be pushed under stringent constraints is useful. In salvage scenarios where conventional options are exhausted, even firmly negative results from conservative single- or dual-function MB systems would be informative. Conversely, if reliable theranostic platforms can deliver molecules excluded from systemic therapy due to toxicity, they could modestly expand antibiotic salvage therapy.
